# RhB@MOF-5 Composite Film as a Fluorescence Sensor for Detection of Chilled Pork Freshness

**DOI:** 10.3390/bios12070544

**Published:** 2022-07-20

**Authors:** Jingyi Li, Ning Zhang, Xin Yang, Xinting Yang, Zengli Wang, Huan Liu

**Affiliations:** 1 College of Food Science and Nutritional Engineering, China Agricultural University, Beijing 100083, China; lijingyi@cau.edu.cn (J.L.); yangxincyn@163.com (X.Y.); 2 Research Center of Information Technology, Beijing Academy of Agricultural and Forestry Sciences, Beijing 100097, China; xingtingyang@nercita.org.cn; 3 National Engineering Research Center for Information Technology in Agriculture, Beijing 100097, China; 4 National Engineering Laboratory for Agri-Product Quality Traceability, Beijing 100097, China; 5 Beijing Key Laboratory of Flavor Chemistry, Beijing Technology and Business University (BTBU), Beijing 100048, China; zh_ningts@btbu.edu.cn

**Keywords:** metal–organic frameworks, rhodamine B, volatile compounds, fluorescence sensing, chilled pork, freshness

## Abstract

This study presents a novel composite thin film based on rhodamine B encapsulated into MOF-5 (Metal Organic Frameworks) as a fluorescence sensor for the real-time detection of the freshness of chilled pork. The composite film can adsorb and respond to the volatile amines produced by the quality deterioration of pork during storage at 4 °C, with the fluorescence intensity of RhB decreasing over time. The quantitative model used for predicting the freshness indicator (total volatile base nitrogen) of pork was built using the fluorescence spectra (excited at 340 nm) of the RhB@MOF-5 composite film combined with the partial least squares (PLS) algorithm, providing R_c_^2^ and R_p_^2^ values of 0.908 and 0.821 and RMSEC (root mean square error of calibration) and RMSEP (root mean square error of prediction) values of 3.435 mg/100 g and 3.647 mg/100 g, respectively. The qualitative model established by the partial least squares discriminant analysis (PLS-DA) algorithm was able to accurately classify pork samples as fresh, acceptable or spoiled, and the accuracy was 86.67%.

## 1. Introduction

Pork is a highly nutritious food rich in protein and several essential amino acids and vitamins ideal for human consumption. However, fresh pork is a perishable food and can easily deteriorate under the action of enzymes and microorganisms in cold-chain logistics, which affects food safety [1]. Traditionally, the freshness of chilled pork has been evaluated based on chemical and microbial indicators, such as the total volatile base nitrogen (TVB-N) value and total viable count (TVC) [2]. However, these traditional detection methods have the disadvantages of being time-consuming and requiring cumbersome pretreatments, high operational requirements, and sample destruction. When meat deteriorates, volatile compounds are produced due to protein degradation and lipid oxidation. Therefore, if these volatile gases can be identified, pork freshness can be monitored in real time.

Electronic nose technology, which is often used to analyze food odors and can compensate for the deficiencies caused by the human subjectivity inherent in sensory evaluation, has been used to determine meat freshness [3,4]. However, as metal oxide sensors in the electronic nose often require the stimulation of electron transfer at a high temperature to produce a gas-sensitive response, these can easily be affected by environmental factors, limiting their application [5]. Cold-chain logistics are now developing from using traditional static methods for detecting food quality to using the “Internet of Things” to provide comprehensive, real-time monitoring [6]. Electronic nose devices are large and expensive, making the rapid sharing of information difficult. More recently, gas sensors based on pigments, which can perform detection and analysis based on the color change of pigments (such as anthocyanin and bromophenol blue) caused by changes in the pH of volatile substances arising in meat, have been developed [7,8,9]. However, these gas sensors have some disadvantages [10], such as pigment instability, a short shelf-life, and low sensitivity. Therefore, developing a rapid and reliable method based on new synthetic gas-sensing materials for identifying the volatile compounds of pork during chilled storage in real time, thus providing a freshness detection technology, has great significance and wide prospects for application in food preservation [11].

Metal–organic frameworks (MOFs) are a type of inorganic, porous, and coordinated polymer material formed from a central metal component and rigid ligands. These highly ordered MOFs are characterized by superior performance resulting from their diversified structure, ultra-high porosity, good adsorption, and surface modifiability, which can play a role in preconcentrating gas molecules and providing sufficient contact with analytes [12,13,14]. With their unique cage and channel-type porous structures, MOFs can efficiently assemble molecules with identifiable characteristics. Furthermore, owing to their greater specific surface area, these can provide a greater number of active sites, leading to more sensitive chemical sensing [15,16]. One type of MOF, Zn_4_(μ_4_-O)-(μ_4_-4-carboxyl-3,5-dimethyl-4-carboxyl-pyrazole)_3_, containing carboxyl groups and coordinated unsaturated metal sites, can selectively capture harmful volatile organic compounds (VOCs) such as sarin and mustard gas [17]. Koh et al. designed a plasmonic nose based on an MOF-encapsulated Ag nanocube array that was able to identify and quantify several harmful VOCs and polycyclic aromatic hydrocarbons (PAHs) at the ppm level [18].

Introducing guest molecules or units, such as dye molecules or chromophores, into MOFs can improve their fluorescence properties, allowing them to be used as a luminous platform for the detection of target substances [19,20]. Yassine et al. used thin films of a rare earth metal (RE)-based MOF with an fcu topology as a fluorescence sensor to detect H_2_S at room temperature [21]. The innovative material Rho@CZJ-3 (a luminescent MOF material containing 1D nanotube channels) was synthesized to probe different volatile organic molecules (VOMs) by modulating the energy transfer efficacy between two different emissions [22]. Combining the highly sensitive and selective identification offered by fluorescence spectroscopy with the gas adsorption properties of MOFs shows potential for producing new gas-sensing materials for practical applications.

**Scheme 1:**
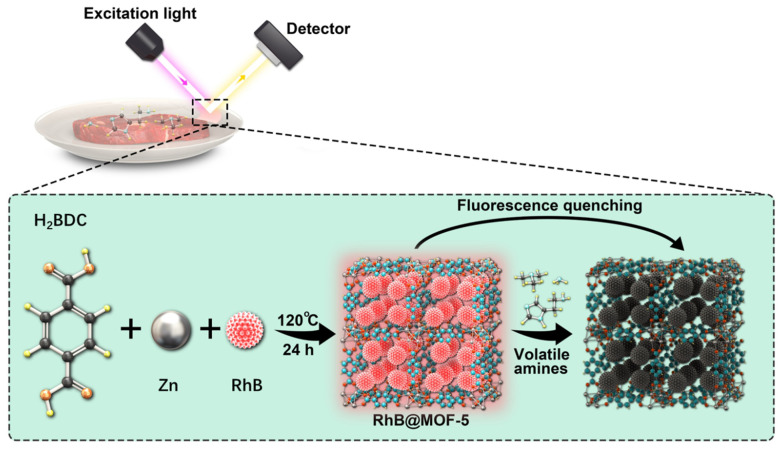
Schematic illustration of the RhB@MOF-5 composite film for pork freshness detection.

This study aimed to synthesize a luminescent rhodamine B (RhB)@MOF-5 gas-sensing thin film for the real-time detection of the freshness of chilled pork (Scheme 1). First, RhB@MOF-5 was prepared from a mixture of zinc nitrate hexahydrate and terephthalic acid in a 2:1 ratio by adding RhB at a concentration of 2 × 10^−3^ mol/L, and the composite film was manufactured by mixing RhB@MOF-5 with polyvinylidene fluoride (PVDF). Next, changes in the volatile components during pork deterioration were analyzed using electronic nose technology, and the response of RhB@MOF-5 to these volatile compounds was discussed. Finally, the relationship between the fluorescence properties of the RhB@MOF-5 composite films and the TVB-N value of pork during storage at 4 °C was determined, and these data, in combination with chemometrics algorithms, were used to establish quantitative and qualitative models for the evaluation of pork freshness.

## 2. Materials and Methods

### 2.1. Materials

Pork samples of *longissimus dorsi* muscles were obtained from the Beijing Ershang Dahongmen Meat Food Co., Ltd. (Beijing, China). The muscles were divided into approximately 50 g pieces on a sterile surface and placed on a tray. The RhB@MOF-5 composite films were made into uniform size labels, numbered, and then attached to plastic wrap on the tray. Thirty prepared pork samples were stored in an incubator at 4 °C.

Zinc nitrate hexahydrate (Zn(NO_3_)_2_·6H_2_O), 1,4-benzenedicarboxylic acid (H_2_BDC), *N,N*-dimethylformamide (DMF), and rhodamine B (RhB) were purchased from Aladdin Reagent Company (Shanghai, China).

### 2.2. Preparation of RhB@MOF-5 Composite Film

RhB (38.3 mg) was added to dimethyl formamide (DMF, 40 mL) containing Zn(NO_3_)_2_·6H_2_O (2.00 mmol) and H_2_BDC (1.00 mmol),and treated with ultrasound for 60 min. The reaction mixture was then transferred to a Teflon-lined reactor and heated in an oven at 120 °C for 24 h. The mixture was then cooled to room temperature and separated by centrifugation (2500 rpm, 5 min), and the products were washed repeatedly with DMF until the supernatant exhibited no fluorescence. After further centrifugation, the products were dried under vacuum at 120 °C for 12 h to obtain the MOF powder [23]. The MOF and PVDF powders were mixed in a 4:5 ratio and then DMF was added. After dispersion using ultrasound for 30 min, the mixture was stirred overnight with a magnetic stirrer to obtain a light pink uniform viscous liquid. This liquid was poured onto a tray in a thin layer and then placed in an oven at 140 °C for about 30 min. After cooling naturally, the films were cut into 1 cm square pieces with a thickness of 0.1 mm and then placed in a dryer.

### 2.3. Instrumentation

Scanning electron microscopy (SEM) images were obtained using the FEI Inspect F50 scanning electron microscope. Powder X-ray diffraction (PXRD) patterns were created using the Panalytical X-ray Diffractometer Smartlab-9 kW. Fourier transform infrared (FTIR) spectra were investigated using a Thermo Scientific Nicolet IS5 FTIR spectrophotometer within a range of 4000 to 400 cm^−1^. The fluorescence spectra were obtained using a HITACHI F-7000 fluorescence spectrophotometer with a solid sample holder enabling the non-destructive analysis. Electronic nose detection was performed using the AlphaMOS Fox 4000 electronic nose with the test parameters as follows: balance temperature of the sample, 37 °C; balance time, 600 s; temperature of the sampler needle, 47 °C; clean and dry air used as the carrier gas at a flow rate of 150 mL/min; sampler volume, 1500 µL; delay time, 10 min; and five samples being processed at one time. The maximum response value of the sensor was taken as the characteristic value.

### 2.4. Storage Stablity Experiment

The RhB@MOF-5 composite films prepared in Section 2.2 were placed in a dark environment at 4 °C. Then, the fluorescence data were collected on day 0, day 30, day 45 and day 60. The fluorescence intensity on day 0 was *I*_0_, and the ratio between day *n* and day 0 was *I_n_/I*_0_.

### 2.5. Standard Measurement of Freshness Indicator

According to the Chinese national standard (GB 5009.228-2016), the TVB-N value can be used as an indicator of meat freshness based on the collection of alkaline volatile nitrogen-containing substances, such as ammonia and amine [24]. Using the Kjeldahl method, the TVB-N value of chilled pork samples was determined on days 1, 2, 3, 4, 6, 8, 11, 13, 15, and 17 of storage, with three pork samples used for each determination.

### 2.6. Statistical Analysis

The data were analyzed using IBM SPSS Statistics (Version 26, IBM Corp., Armonk, NY, USA) and graphs were plotted using Origin 2018 (OriginLab Corp., Northampton, MA, USA).

Partial least squares (PLS) regression was used to identify quantitative correlations between the spectral data and the freshness indicator, using Unscrambler X 10.0.1 (CAMO PROCESS AS, Oslo, Norway). The performance of the models was assessed using the square of the correlation coefficient (R^2^), the root mean square error of calibration (RMSEC), and the root mean square error of prediction (RMSEP). The models will generally give higher R_c_^2^ and R_p_^2^ values and lower RMSEC and RMSEP values. When R^2^ is closer to 1 and the R_c_ and R_p_ values are more similar, the predictions made by the models are usually more accurate [25,26].

Partial least squares discriminant analysis (PLS-DA) is a linear classification method. It can combine the characteristics of PLS regression with the discrimination ability of a classification technique and is used to find mathematical models that can identify which class each sample belongs to [27]. PLS-DA models were applied using SIMCA-P 11.5 (Umetrics, Umeå, Sweden), which allowed the graphical visualization and understanding of the different data patterns and relationships using the scores and loadings of latent variables (LVs).

## 3. Results and Discussion

### 3.1. Synthesis and Characterization of RhB@MOF-5

#### 3.1.1. Selection of RhB Concentration

**Figure 1:**
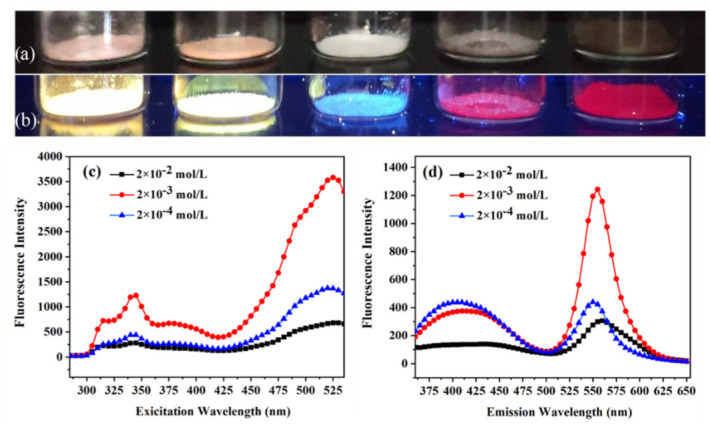
(**a**) Images of RhB@MOF-5 (RhB at a concentration of 2 × 10^−3^ mol/L), RhB@MOF-5 (RhB at a concentration of 2 × 10^−2^ mol/L), MOF-5, RhB mixed with MOF-5 and RhB under visual light. (**b**) Images of RhB@MOF-5 (RhB at a concentration of 2 × 10^−3^ mol/L), RhB@MOF-5 (RhB at a concentration of 2 × 10^−2^ mol/L), MOF-5, RhB mixed with MOF-5 and RhB under 365 nm UV light. (**c**) Fluorescence spectra (emission at 550 nm) for RhB@MOF-5 (RhB at concentrations of 2 × 10^−2^, 2 × 10^−3^, and 2 × 10^−4^ mol/L). (**d**) Fluorescence spectra (excitation at 340 nm) for RhB@MOF-5 (RhB at concentrations of 2 × 10^−2^, 2 × 10^−3^, and 2 × 10^−4^ mol/L).

Images of MOF-5, RhB, RhB mixed with MOF-5, and RhB@MOF-5 with different RhB concentrations under visual light and 365 nm UV light are shown in Figure 1a,b. The luminescence intensity of RhB@MOF-5 was much higher than that of pure RhB and MOF-5 powder. Fluorescent dyes often undergo fluorescence quenching due to aggregation, but the porous structure of MOFs can act as a “solid solvent” [19]. According to the related reference [28], the pore size of MOF-5 is around 1.56 nm. The immobilization of dyes into the pore spaces of MOFs can disperse the dye molecules and minimize aggregation-induced quenching, resulting in increased luminescence. Furthermore, the photoluminescence properties of RhB@MOF-5 were clearly different from those of the mixture of MOF-5 and RhB, which indicated that RhB had been enclosed in the pores of MOF-5.

Previous research has shown that the fluorescence emission wavelength of RhB is about 550 nm [29]. To determine the optimal RhB concentration, fluorescence spectra of RhB@MOF-5 powder samples with different RhB concentrations (including 2 × 10^−2^, 2 × 10^−3^, and 2 × 10^−4^ mol/L) were measured with emission at 550 nm, as shown in Figure 1c. The fluorescence intensities exhibited maximum values when the RhB concentration was 2 × 10^−3^ mol/L. When the dye concentration is low, the fluorescence intensity increases with increasing dye concentration. However, when the dye concentration reaches a certain value, its aggregation will be enhanced, which increases the nonradiative energy transfer between molecules but decreases the fluorescence intensity [30]. MOF-5 provides abundant pores that allow RhB to be well dispersed, which decreases aggregation and thus improves the fluorescence properties. Figure 1d also shows the redshift in the emission wavelength as the concentration of RhB increased. This might be due to energy transferring between the component molecules when RhB aggregated into a dimer or polymer form. Therefore, the RhB/DMF solution with a concentration of 2 × 10^−3^ mol/L was selected to prepare RhB@MOF-5 for the following experiments.

#### 3.1.2. Characterization of RhB@MOF-5

**Figure 2:**
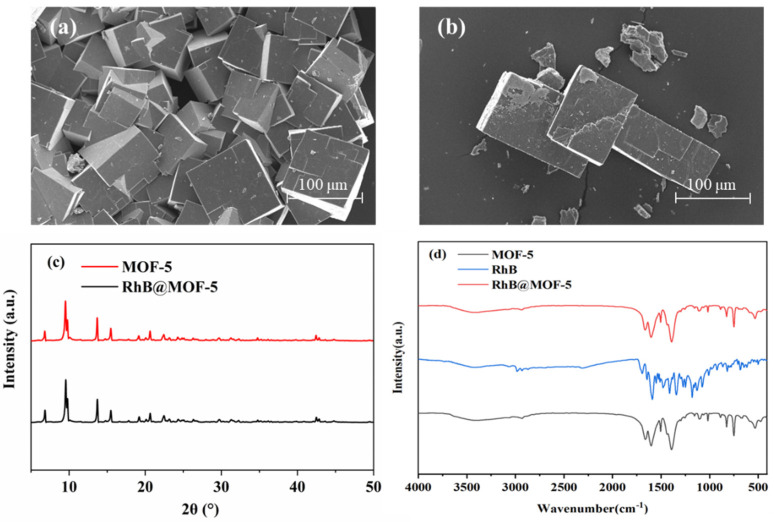
(**a**) SEM image of MOF-5; (**b**) SEM image of RhB@MOF-5; (**c**) PXRD patterns of MOF-5 and RhB@MOF-5; and (**d**) FT-IR spectra of MOF-5, RhB and RhB@MOF-5.

The morphological and structural characterization of RhB@MOF-5 was conducted using the following tests. First, SEM images of MOF-5 and RhB@MOF-5 were analyzed, as shown in Figure 2a,b. The size of MOF-5 was 60–80 μm, while the size of RhB@MOF-5 was about 80 μm. The surface of RhB@MOF-5 was slightly rougher than that of MOF-5. RhB@MOF-5 is a square crystal with a similar structure to that of MOF-5, indicating that RhB encapsulation did not change the morphology of MOF-5. The powder X-ray diffraction (PXRD) characterization of RhB@MOF-5 was also conducted, as shown in Figure 2c. This showed that RhB encapsulation did not obviously change the diffraction peak position or reduce the intensity of MOF-5. Therefore, RhB@MOF-5 retained the structure of MOF-5. Moreover, the XRD peaks of MOF-5 and RhB@MOF-5 were similar, indicating that RhB molecules had been enclosed in the pores rather than being physically adsorbed on the surface of MOF-5, and that the composite material had been successfully prepared. Figure 2d shows the FT-IR spectra of MOF-5 and RhB@MOF-5, which were highly similar, with no shift in the symmetric and asymmetric stretching vibrations of carboxylic acid at 1598 and 1390 cm^−1^, respectively, indicating that RhB encapsulation did not affect the coordination between H_2_BDC and zinc(II) ions.

### 3.2. Response of RhB@MOF-5 to Volatile Compounds during Pork Deterioration

#### 3.2.1. Analysis of Characteristic Volatile Components by Electronic Nose

**Figure 3:**
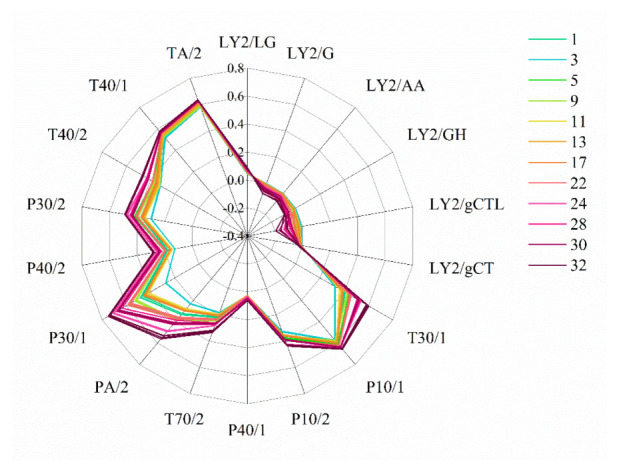
Changes in the response of 18 sensors of the electronic nose to pork samples stored at 4 °C (day 1, 3, 5, 9, 11, 13, 17, 22, 24, 28, 30 and 32).

The electronic nose is an odor analysis system based on a gas sensor array, with the different sensors able to identify the various volatile components. Figure 3 shows the changes in the responses of the 18 sensors of the electronic nose to volatile compounds from pork stored at 4 °C (Table S1). As the storage time increased, the response values of the electronic nose sensors also changed, with sensors LY2/G, LY2/AA, LY2/GH, and LY2/gCTL showing particularly obvious changes. Sensor LY2/G responded to changes in ammonia, amine compounds, ketones and alcohols; sensor LY2/AA responded to changes in ethanol, acetone, and ammonia; sensor LY2/GH responded to changes in ammonia and amine compounds; and sensor LY2/gCTL responded to changes in H_2_S. Overall, this showed that the contents of compounds including ammonia, amines, ketones, and alcohols changed a great deal as pork deteriorated during chilled storage.

#### 3.2.2. Infrared Analysis of RhB@MOF-5 Response to Pork Deterioration

**Figure 4:**
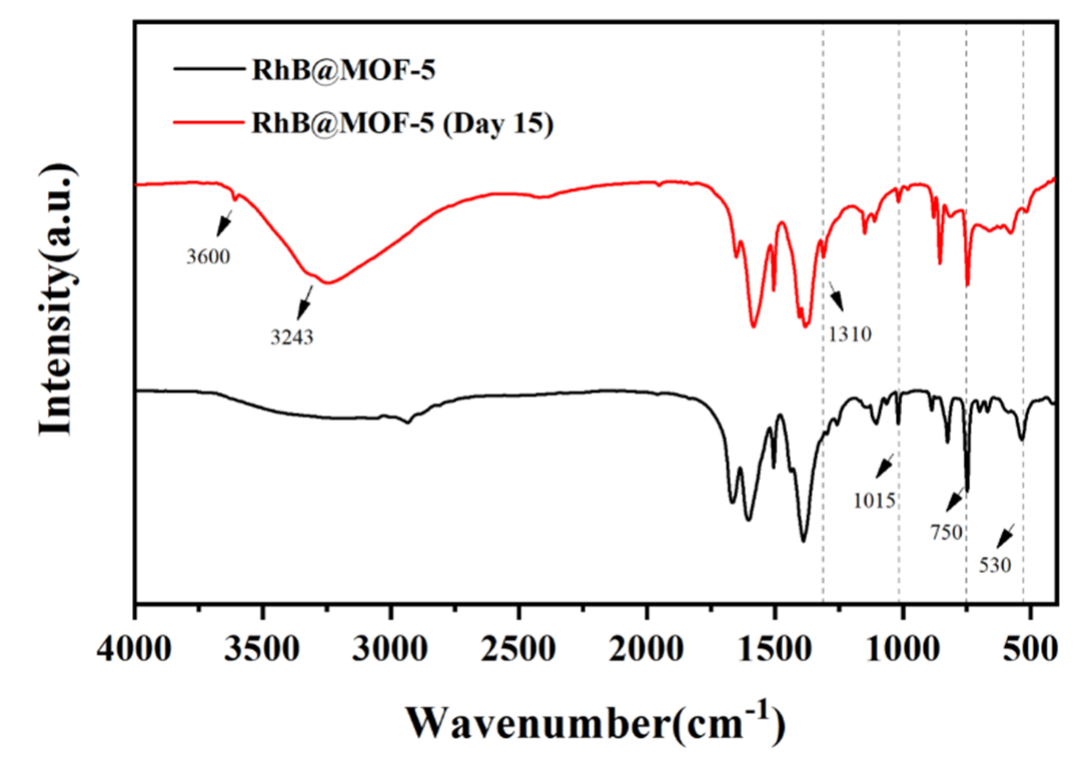
FT-IR spectra of RhB@MOF-5 response to pork deterioration.

Infrared analysis can be used to characterize the chemical bonds and functional groups of substances to provide specific fingerprinting characteristics. FT-IR spectra of RhB@MOF-5 were collected after the adsorption of volatile components produced by pork deterioration, as shown in Figure 4. Characteristic bands of the symmetric and asymmetric stretching vibrations of terephthalic acid were still present at 1598 and 1390 cm^−1^, respectively. The intensity of the Zn–O band at 530 cm^−1^, the C-H band at 750 cm^−1^ and the C=O band at 1015 cm^−1^ decreased, showing that the structure of RhB@MOF-5 had been affected. A distinct, wide band appeared from 2800 to 3500 cm^−1^ and a sharp band appeared at 3600 cm^−1^, which were both attributed to O-H stretching, showing that RhB@MOF-5 had absorbed water. Furthermore, a N-H stretching vibration band was observed at 3243 cm^−1^ and a C-N stretching vibration band appeared at 1310 cm^−1^, which indicated that RhB@MOF-5 adsorbed volatile amines produced by the quality deterioration of pork during storage.

#### 3.2.3. Fluorescence Sensing Analysis of RhB@MOF-5 Response to Pork Deterioration

**Figure 5:**
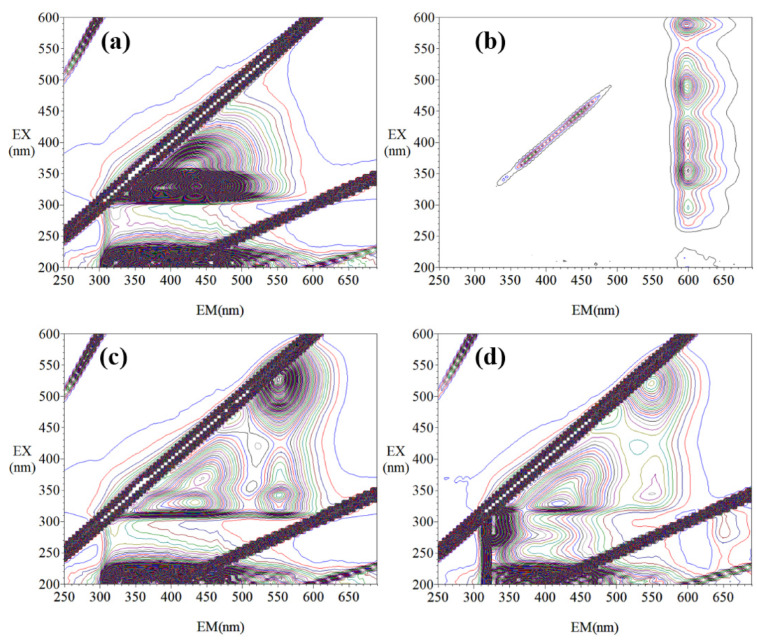
(**a**) Three-dimensional fluorescence spectrum of MOF-5; (**b**) 3-D fluorescence spectrum of RhB; (**c**) 3-D fluorescence spectrum of RhB@MOF-5; and (**d**) 3-D fluorescence spectrum of RhB@MOF-5 response to pork deterioration.

Three-dimensional fluorescence spectra can provide comprehensive information on components in a complex system. Four fluorescence peaks were identified in the 3-D florescence spectra of RhB@MOF-5, as shown in Figure 5, Ex/Em peaks located at 330 nm/435 nm and 365 nm/440 nm were attributed to MOF-5, while Ex/Em peaks of 340 nm/550 nm and 520 nm/550 nm were characteristic peaks of RhB. As shown in Figure 5d, when RhB@MOF-5 adsorbed volatile compounds from pork deterioration, the fluorescence intensities of the two characteristic peaks of RhB (340 nm/550 nm and 520 nm/550 nm) greatly decreased. The RhB molecules were distributed in the pores of MOF-5 mainly through electrostatic action. When volatile amines diffused and adsorbed into the pores of MOF-5 and made contact with RhB, the electron cloud density of RhB molecules changed, resulting in a decrease in fluorescence intensity. These results demonstrated the feasibility of using RhB@MOF-5 as a gas-sensing material for monitoring the quality deterioration and detecting the freshness of chilled pork.

### 3.3. Application of RhB@MOF-5 Composite Film to Detecting Chilled Pork Freshness

**Figure 6:**
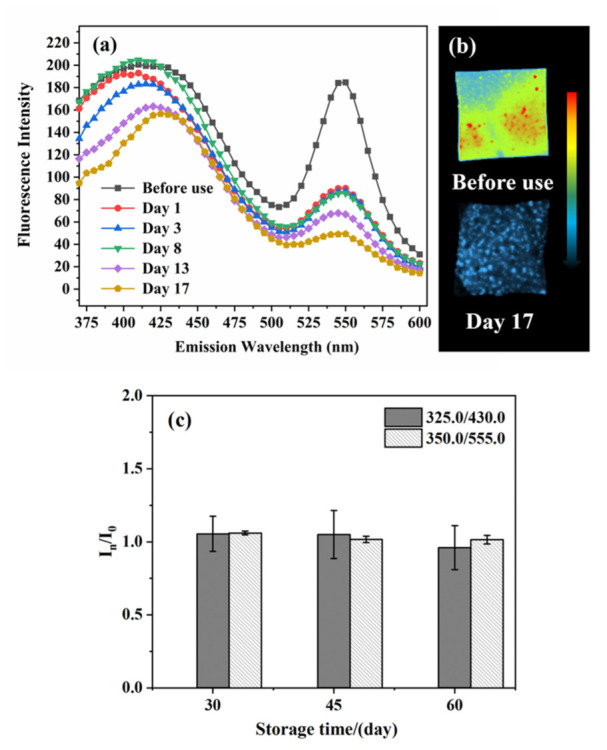
(**a**) Fluorescence emission spectra (excitation at 340 nm) of RhB@MOF-5 composite films of pork samples stored at 4 °C. (**b**) Fluorescence image of RhB@MOF-5 composite film before and after pork spoilage (excitation light of 530 nm). (**c**) Storage stability of RhB@MOF-5 composite film at 4 °C in dark conditions.

To broaden the applications of MOFs and weaken the effect of their inherent vulnerability, a composite film was prepared using RhB@MOF-5 and PVDF powders at a 4:5 ratio (*w/w*) using the mixed matrix method, which simultaneously provided the mechanical flexibility of a polymer matrix and the high porosity of MOFs. A storage test showed that the composite film maintained its fluorescence stability for more than 60 d in a dark environment at 4 °C, as shown in Figure 6c.

Fluorescence wavelength scanning using a fixed excitation or emission wavelength has the advantage of simplifying spectral analysis and reducing the influence of light scattering, and only takes a few seconds per sample (3-D fluorescence spectroscopy requires more than 5 min for each scan). As shown in Figure 5c, scanning the RhB@MOF-5 composite film at an excitation wavelength of 340 nm facilitated simultaneous observation of the changes in characteristic peaks of both the MOF-5 (Ex/Em at 330 nm/435 nm) and RhB (Ex/Em at 340 nm/550 nm), allowing the properties of RhB@MOF-5 to be comprehensively determined. Figure 6a shows the fluorescence emission spectra of RhB@MOF-5 composite films (excited at 340 nm) during pork storage at 4 °C. Two characteristic peaks were identified at emission wavelengths near 420 nm and 550 nm, which were associated with MOF-5 and RhB, respectively. The intensities of these two peaks decreased significantly with increasing storage time. Furthermore, Figure 6b shows the fluorescence images of composite RhB@MOF-5 films (under excitation light of 530 nm) before and after pork spoilage, showing that the fluorescence intensity had clearly decreased. Overall, the volatile compounds produced by pork deterioration greatly affected the fluorescence properties of the composite film.

**Figure 7:**
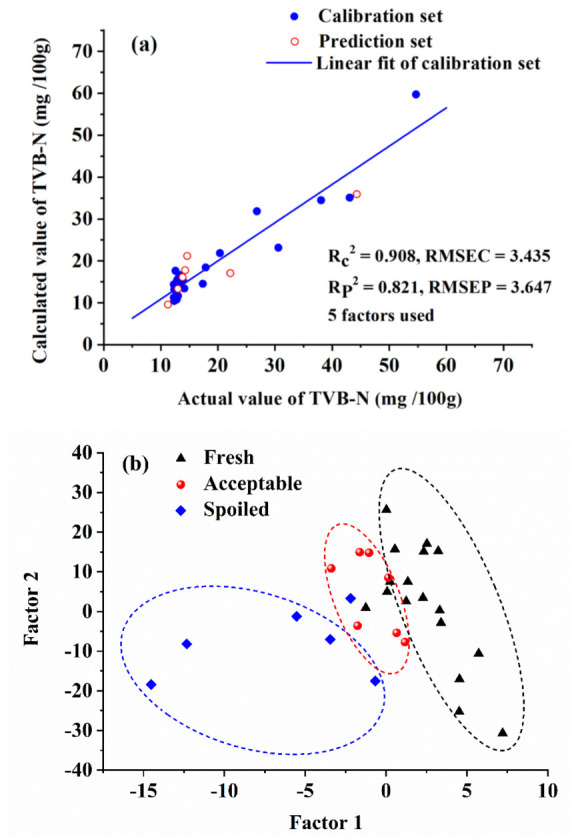
(**a**) The results of PLS modeling of the TVB-N values determined for pork samples during chilled storage. (**b**) The latent variable scores of PLS-DA modeling of pork samples (classified as fresh, acceptable or spoiled).

The TVB-N value can be used as an indicator of meat freshness based on the collection of alkaline volatile nitrogen-containing substances, such as ammonia and amine. The TVB-N values of the pork samples were measured on days 1, 2, 3, 4, 6, 8, 11, 13, 15, and 17 of chilled storage at 4 °C, and ranged between 11.17 and 54.68 mg/100 g, with an average value of 19.18 mg/100 g. Partial least squares (PLS) regression was used to establish a quantitative model to identify the relationship between the fluorescence spectral data of RhB@MOF-5 composite films and the TVB-N values of pork samples. The data matrix from 30 pork samples was randomly divided into a calibration set and a prediction set at a ratio of ~3:1 (23 in the correction set and 7 in the prediction set). Figure 7a shows the quantitative modeling results, with R_c_^2^ and R_p_^2^ values of 0.908 and 0.821, respectively, and RMSEC (root mean square error of calibration) and RMSEP (root mean square error of prediction) values of 3.435 and 3.647 mg/100 g, respectively. With both the R_c_^2^ and R_p_^2^ values being greater than 0.8, and the RMSEC and RMSEP values being similar, the PLS regression model was considered to be effective with a good prediction accuracy.

According to Chinese national food safety standards for meat quality, the TVB-N values of fresh pork is below 15.0 mg/100 g, of acceptable pork is around 15.0–25.0 mg/100 g, and of spoiled pork is above 25.0 mg/100 g [24], which serves as a reference for establishing a qualitative model based on fluorescence spectra of RhB@MOF-5 film combined with the PLS-DA algorithm. Figure 7b shows the visualization of the latent variable score plot map of the qualitative model, as defined by the principal components 1 and 2, and the three classes of pork freshness (fresh, acceptable and spoiled) can be clearly distinguished with an accuracy of 86.67%, which can be used to estimate the freshness classifications of pork samples reliably.

Overall, the RhB@MOF-5 composite film has the potential to be used as a fluorescence gas sensor for the sensitive, rapid, and nondestructive detection and visual monitoring of pork quality deterioration and freshness during refrigerated storage.

## 4. Conclusions

This study developed a RhB@MOF-5 composite thin film that can be used as an intelligent label in pork packaging, enabling the sensitive and nondestructive detection of pork freshness during chilled storage based on fluorescence sensing. Changes in the FT-IR spectra of RhB@MOF-5 after exposure to deteriorating pork showed that this material adsorbed volatile amines. The fluorescence intensity of the RhB@MOF-5 composite film tended to weaken with increasing storage time. Quantitative models for predicting the freshness indicator and qualitative models for the classification of pork freshness both produced satisfactory results. This study provides the first examination of the dye@MOF system as a gas-sensing material for meat freshness evaluation, showing its potential for application in food quality detection.

## Data Availability

The data presented in this study are available upon request from the corresponding author.

## Supplementary Materials

The following supporting information can be downloaded at: https://www.mdpi.com/article/10.3390/bios12070544/s1, Table S1: Eighteen channels in electronic nose.
