# Supplementary material for: RhB@MOF-5 Composite Film as a Fluorescence Sensor for Detection of Chilled Pork Freshness
Source: Biosensors (Basel). 2022 Jul 20;12(7):544. doi: 10.3390/bios12070544 (PMC9313163; doi:10.3390/bios12070544)
Supplement: Supplementary file 1 [file biosensors-12-00544-s001.zip › biosensors-1807103-supplementary.pdf]

Table S1. Eighteen channels in electronic nose

Channel	Sensitive substance
LY2/LG	Chlorine, fluorine, nitrogen oxides, sulfides
LY2/G	Ammonia, amines, ketones, alcohols
LY2/AA	Ethanol, acetone, ammonia
LY2/GH	Ammonia, amine compounds
LY2/gCTL	Hydrogen sulfide
LY2/gCT	Propane, butane
T30/1	Polar compounds, hydrogen chloride
P10/1	Ammonia, chlorine compounds, acids
P10/2	Methane, ethane
P40/1	Fluorine, chlorine
T70/2	Aromatic compounds
PA/2	Ethanol, ammonia and amines
P30/1	Flammable gas detection
P40/2	Chloride
P30/2	Combustion gas detection
T40/2	Chloride
T40/1	Fluoride
TA/2	General air pollution detection, alcohol
